# Digital technology and its impacts on the sleep quality and academic performance during the pandemic

**DOI:** 10.1055/s-0042-1755395

**Published:** 2022-12-19

**Authors:** Luana Guimarães Lima Cabral, Taynara Nunes Queiroz, Laércio Pol-Fachin, Adriana Rodrigues Libório dos Santos

**Affiliations:** 1 Centro Universitário CESMAC, Faculdade de Medicina, Maceió AL, Brazil.; 2 Instituto Estadual do Cérebro Paulo Niemeyer, Departamento de Neurocirurgia, Rio de Janeiro RJ, Brazil.

**Keywords:** Students, Medical, Circadian Rhythm, Academic Performance, Digital Technology, Emergency Remote Teaching, Estudantes de Medicina, Ritmo Circadiano, Desempenho Acadêmico, Tecnologia Digital, Ensino Remoto de Emergência

## Abstract

**Background** Sleep deficits caused by the overuse of digital technology is observed among medical students. Due to the coronavirus disease 2019 (COVID-19) pandemic, an emergency remote teaching method was put into practice, which may have resulted in changes in the sleep-wake cycle. The balance between the influences of external and internal synchronizers can be affected by sudden alterations in daily life, including changes in nightly habits and sleep quality, which can lead to increased levels of anxiety and reduced functional performance, for example.

**Objective** To understand the relationship between the use of digital technology, changes in the circadian cycle, and academic performance during the pandemic.

**Methods** The present is an analytical, cross-sectional, observational study in which a sample of 123 medical students filled out an online questionnaire on self-perception regarding sleep quality and academic performance before and during the pandemic.

**Results** Assessing changes in sleep quality and productivity, the study revealed that 100% of the students made continuous use of screens before bedtime. Thus, during the period of social distancing and remote classes, 77.2% of the students reported “poor” or “very poor academic performance, which was probably related to the fact that 65.9% of these students were unable to maintain their productivity due to daytime sleepiness.

**Conclusions** The prolonged use of screens was associated with poor sleep quality and changes in academic performance, with significant psychological impact. Thus, it is worth emphasizing the importance of sleep hygiene in light of the new forms of teaching implemented during the COVID-19 pandemic.

## Resumo

**Introdução** Observa-se um déficit de sono ocasionado pelo uso excessivo de tecnologias digitais entre estudantes de medicina. Em face da pandemia da doença do coronavírus 2019 ( *coronavirus disease 2019* , COVID-19, em inglês), um método de ensino à distância foi adotado, e pode ter acarretado mudanças no ciclo de sono e vigília. O equilíbrio entre as influências dos sincronizadores externos e internos pode ser afetado por mudanças bruscas na vida diária, isto inclui alterações nos hábitos noturnos e na qualidade do sono, que podem causar aumento dos níveis de ansiedade e redução do desempenho funcional.

**Objetivo** Compreender a relação entre o uso de tecnologias digitais, alterações no ciclo circadiano, e desempenho acadêmico durante a pandemia.

**Métodos** Trata-se de um estudo observacional, analítico e transversal, no qual uma amostra de 123 estudantes de medicina responderam a um questionário *online* sobre a autopercepção referente à qualidade do sono e ao desempenho acadêmico antes e durante a pandemia.

**Resultados** Com a avaliação das alterações na qualidade do sono e na produtividade, o estudo revelou que 100% dos alunos faziam uso contínuo de telas antes do horário de dormir. Assim, durante o período de distanciamento social e aulas remotas, 77,2% dos alunos relataram que o desempenho acadêmico era “ruim” ou “péssimo”, o que provavelmente estava relacionado ao fato de que 65,9% desses alunos que não conseguiram manter sua produtividade por conta de sonolência diurna.

**Conclusões** O uso de telas por tempo prolongado foi associado a mudanças relacionadas à baixa qualidade do sono e a mudanças no desempenho acadêmico, com impacto psicológico significativo. Assim, vale ressaltar a importância da higiene do sono diante das novas formas de ensino implantadas durante a pandemia da COVID-19.

## INTRODUCTION

Human beings present biological rhythms during the 24 hours of the day. One of the most important is the sleep-wake cycle, which is externally influenced by synchronizers such as light stimuli, personal habits, and social factors. 1 In addition to these external stimuli, endogenous factors, such as the levels of melatonin and cortisol, play an important role in the functioning of the sleep-wake cycle. Melatonin is centrally involved in antioxidation, maintenance of circadian rhythmicity, sleep regulation, and neuronal survival. Cortisol, in turn, is responsible for the waking state during the day. 1

The absence of light during nighttime causes changes in the retinal cells, which are responsible for the photoreception and transduction of the light stimulus transmitted via glutamate through the retinohypothalamic tract to the suprachiasmatic nucleus, and this, in turn, causes the upper cervical ganglion to release the noradrenaline neurotransmitter, with consequent production and release of melatonin by the pineal gland. 2 Physiologically, studies 3 show that exposure to self-luminous electronic displays before bedtime may cause statistically significant suppression of melatonin, as well as an increase in the sleep latency and a reduction in the duration of rapid-eye-movement (REM) sleep, which is responsible for the preservation of cognitive functions.

Sleep fulfills important needs within the balance-restoring functions of several human biological and social functions, including the learning process, which occurs with memory consolidation. 4 The balance between the influences of external synchronizers and the internal temporal order, represented by endogenous stimuli, can be altered by abrupt changes in daily life. They include changes in nightly habits and sleep quality, which can cause sleep disorders, gastrointestinal changes, mood disorders, anxiety disorders, as well as reduced functional performance. 5

In fact, among undergraduate students, it has been observed 6 that increased bedtime use of mobile phones decreased academic performance and sleep quality. Students already tend to have irregular sleep patterns and suffer from partial sleep deprivation, as academic demands and class schedules increase the need for greater exposure to light and consequent desynchronization of the light-dark cycle and the natural sleep-wake cycle to maintain academic obligations. 5 Thus, recent studies emphasize the coronavirus disease 2019 (COVID-19) pandemic as a modifier of social habits. Cellini et al. 7 emphasized that the different chronotypes of young students made them more susceptible to sleep disorders and, consequently, to increased feelings of anxiety and stress in the face of adversity.

Moreover, the emotional burden caused by the change in the current scenario, associated with the pressure for productivity on the part of undergraduate courses, leads to sleep deprivation and real physiological changes in students, such as impaired executive function and vigilant attention, and increased stressors, which can interfere with cognitive functions such as memory. 8 In this context, the present study aims to understand the relationship involving the use of digital technology, and changes in the circadian cycle and academic performance during the pandemic among medical students from a private institution.

## METHODS

The present is an observational, analytical, cross-sectional study performed at a University Center located in the city of Maceió, state of Alagoas, Northeastern Brazil. We invited medical students to fill out a questionnaire via Google Forms composed of 12 questions, each with a maximum of 5 answer options, about their perception of their sleep quality and academic performance, as well as about the presence of symptoms related to circadian rhythm disorders before the pandemic and during the period they underwent Remote Emergency Teaching, such as daytime sleepiness, delayed sleep onset, and decreased alertness.

The present study followed the ethical standards established by the Brazilian National Ethics in Research Comission (Comissão Nacional de Ética em Pesquisa, CONEP, in Poertuguese), and was approved by the institutional Ethics in Research Committee under CAAE 38758320.8.0000.0039, opinion number 4.379.412. The students were informed through the informed consent form (ICF) that their answers would only be used for the preparation of data associated with the research project. After agreeing with all the terms presented, the questions were released to be answered.

The sample consisted of 123 medical students enrolled in the same institution in which the present study was conducted, who were attending classes during the period of Remote Emergency Teaching. Moreover, there were no distinctions of gender or specific age groups. The exclusion criteria were students not enrolled in the School of Medicine during the period analyzed in the study.

The recruitment was performed through the social media platform WhatsApp, and the research project was also publicized through the institutional news board, so that information about it would be available to all medical students.

The data obtained were analyzed using descriptive statistics, followed by analytical statistics, using the two-way Chi-squared test.

## RESULTS

After analyzing and tabulating the data from the questionnaires, we observed that the results were compatible with those of the existing literature in terms of changes in the sleep-wake cycle, learning and productivity due to the influence of external factors such as the use of digital technology, which became more prevalent in this period.

To identify possible changes experienced by the students due to the COVID-19 pandemic and the consequent changes in the teaching-learning model with the use of digital platforms, we analyzed symptoms related to alterations in sleep and in the emotional state, which interfere with the sleep-wake cycle. We observed that 100% of the sample of 123 students made continuous daily use of digital platforms before bedtime, which may be the reason for the rate of 90.2% (111/123) who reported at least 1 symptom related to circadian rhythm disorders, such as an increased level of stress, and other symptoms, such as headache, anxiety, discomfort in vision, and drowsiness during school hours ( Table 1 ). Moreover, between 40% and 66.7% of the participants reported waking up more often than usual during the night throughout the pandemic ( Table 2 ), while 91.1% (112/123) reported that it took them longer to fall asleep, characterizing a discontinuous sleep cycle.

**Table 1:** Occurrence of symptoms related to disturbances in the sleep cycle during the COVID-19 pandemic (n = 123)

Variable		n	%	*p* -value ^a^
**Increased levels of stress**	Yes	58	47.2	< 0.0001
	Quite	49	39.8	
	Not much	16	13.0	
	No	0	0.0	
**Headache or visual discomfort**	Yes	45	36.6	< 0.0001
	Quite	69	56.1	
	Not much	8	6.5	
	No	1	0.8	
** Anxiety ^b^**	Yes	40	32.5	0.0014
	Quite	25	20.3	
	Not much	17	13.8	
	No	12	9.8	
**Drowsiness during school hours**	Yes	51	41.5	< 0.0001
	Quite	30	24.4	
	Not much	33	26.8	
	No	9	7.3	

Notes: ^a^ Two-way Chi-squared test. ^b^ Regarding the symptoms of anxiety, 23,6% (29/123) of students chose not to answer.

**Table 2:** Self-perception of sleep quality during COVID-19 pandemic (n = 123)

Variable		Participants	%	*p* -value ^a^
** Dysregulated sleep ^b^**	Yes	11	8.9	0.0016
	Quite	38	30.9	
	Not much	40	32.5	
	No	34	27.6	
** Discontinuous sleep ^c^**	Yes	36	29.3	0.0029
	Quite	46	37.4	
	Not much	15	12.2	
	No	26	21.1	
**More time required until sleep onset**	Yes	30	24.4	0.0001
	Quite	51	41.5	
	Not much	31	25.2	
	No	11	8.9	

Notes: ^a^ Two-way Chi-squared test. ^b^ In the questionnaire, dysregulated sleep meant a general perception of sleep quality. ^c^ In the questionnaire, discontinuos sleep meant waking up more frequently at night.

We also observed that 6.5% (8/123) of the participants started using melatonin to improve the quality of their sleep during the pandemic, and 3.3% (4/123) of the students were already using it and increased the dose during the pandemic, but 84.6% (104/123) reported that never used melatonin.

Finally, during the period of social distancing and remote classes, 77.2% (95/123) of the students reported that their academic performance was “poor” or “very poor”, which was probably related to the fact that 92.7% (114/123) of them reported that they could not maintain their productivity during the day because of sleepiness/drowsiness. Only 7.3% (9/123) reported they could keep focused during classes all day long, and 47.2% (58/123) could only do it for 1 to 2 hours, reflecting the lack of cognitive functions necessary for memory consolidation.

## DISCUSSION

The present study aims to establish a relationship between changes in the teaching method during the pandemic and their impact on the sleep cycle. The results obtained show that 90.2% of the students reported at least 1 symptom related to circadian rhythm disorders. Around 65% of them reported it took them longer to fall asleep, and that they experienced sleep. In fact, although sleep disruption in medical students is a well-known issue, 9 sleep problems were extensively reported in the general population during the COVID-19 pandemic. 10 11 12 However, there is a need for studies about the effects of sleep deprivation and of the light emitted by electronic devices on the sleep-wake cycle. During the COVID-19 pandemic, students had to adapt to new teaching methods, as the data herein obtained reveal alterations in the attention span during remote classes and increased psychosocial stress related to the new form of adaptation.

The data obtained also highlights the high prevalence of a self-perceived decrease in academic performance. It is known that sleep quality and daytime sleepiness are correlated with the academic grades of medical students. 9 Thus, the rate of 92,8% of drowsiness during school hours and of 40% to 65% of decreased sleep quality observed in the present study do represent high prevalence rates.

In addition to the intrinsic factors that drive organic functions, the constant and prolonged use of digital technology, together with new teaching methods, have important consequences for vision, since the retinal cells, which are responsible for the photoreception of light stimulus, are affected by intense exposure to said stimulus. 1 Despite the divergences in the literature regarding the direct relationship between excessive screen time and the incidence of headache, a research 13 reported that the student's learning process is related to possible visual discomfort, corroborating that they are increasingly interfering in performance and concentration in the face of online studies, where the use of screens for up to 30 minutes was associated with a 95% greater risk of migraine. 13 These findings are in line with those of the present study, in which 99.2% of the students reported episodes of headache and vision disorders ( Table 1 ).

Based on the obtained results, melatonin use cannot be inferred as a direct alternative used by students in the face of changes in the new teaching model, considering the low percentage of its use where 84.6% (104/123) have never used or began to use in face of the pandemic. In addition, despite the low adherence, the natural deregulation of melatonin through long exposure to digital technology also evidences important deprivation and alterations in the quality of sleep, responsible for the increase in stress hormones that reflect on quality of life. 3

It is worth noting that the emotional conditions involved in this whole process, the involvement of the respondents themselves and the uncertainties about the future, led to an increase in anxiety levels, which are a *sine qua non* condition for understanding the phenomenon that led to adaptation from the physiological organism to a new didactic reality. Moreover, comparing the literature and the answers obtained from the students in the present study, we were able to establish a relationship between the students' anxiety levels and their sleep-wake cycle, as the temporal factors of students with anxious personalities have less liability as to the ability to adapt to the new demands. 5 Thus, it is possible to observe the prevalence of symptomatic students during the pandemic period together with the new teaching model, since 90.2% had at least some manifestations arising ( Table 1 ). A high prevalence of anxiety was also observed during the COVID-19 pandemic among the general population in China 14 and Brazil. 10 The combination of endogenous factors, such as anxiety itself and lack of sleep, is highly affected by the exogenous factors investigated in the research, like the previous analyses. 3 The evidenced data resulting from delay for the sleep onset and reduced sleep duration reflect damage to sleep health in up to 90% of young people, such as the low concentration and impairment of daily activities in up to 65.9% of students. 3 Accordingly, in the present study, some degree of difficulty in falling sleep was reported by 91.1% of the students ( Table 2 ), while 92,7% could not keep focused on remote classes all day long ( Table 1 ). Such data reflects the possible deleterious consequences of prolonged exposure to digital technology during the pandemic.

In conclusion, excessive screen time, although a necessity due to the pandemic, was associated with symptoms related to changes in sleep quality, with a decrease in academic performance, as well as increased levels of anxiety and stress. Thus, in the present study, physical symptoms such as daytime drowsiness and decreased attention span were prevalent among medical students attending classes through emergency remote teaching. It is worth emphasizing the importance of sleep hygiene, which is a regulating factor for academic performance and psychological fulfillment considering the new forms of teaching implemented during the COVID-19 pandemic.
